# Supplementary material for: Characterization of a novel zebrafish model of SPEG-related centronuclear myopathy
Source: Dis Model Mech. 2022 May 9;15(5):dmm049437. doi: 10.1242/dmm.049437 (PMC9118044; doi:10.1242/dmm.049437)
Supplement: Supplementary information [file dmm-15-049437-s1.pdf]

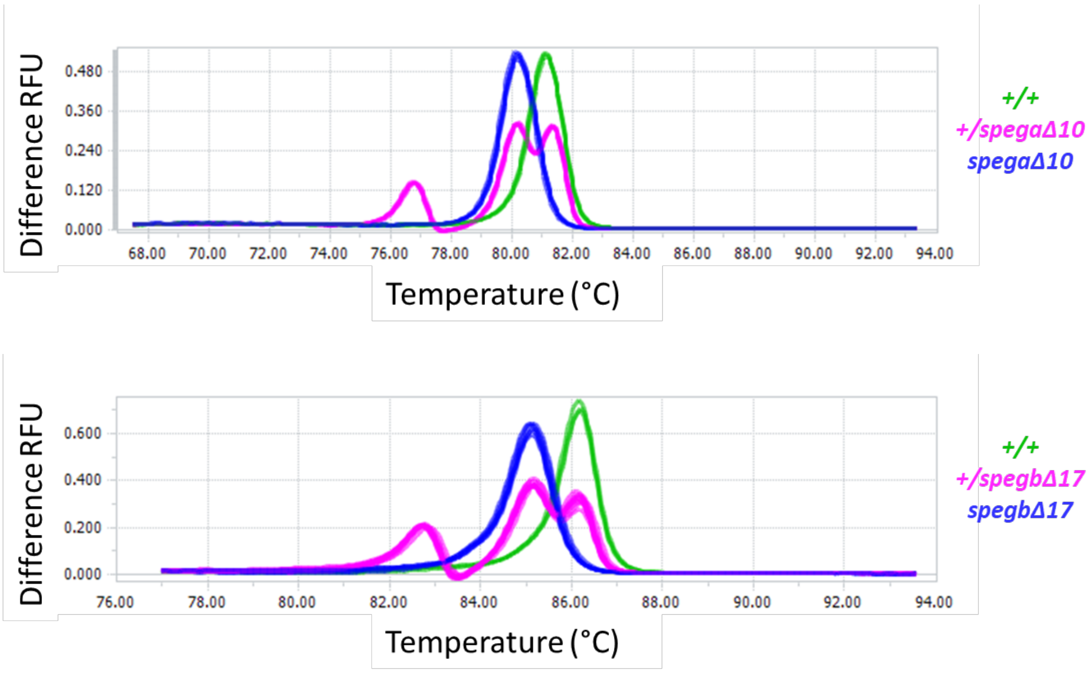

Fig. S1. ***spegaΔ10;spegbΔ17* mutant zebrafish genotyping**.

High Resolution Melting (HRM) analysis was used to genotype *spega* (top) and/or *spegb* (bottom) CRISPR lines. Wildtype (*green*), Heterozygous (*magenta*), and Homozygous mutants (*blue*). RFU: relative fluorescence units.

Disease Models & Mechanisms • Supplementary information

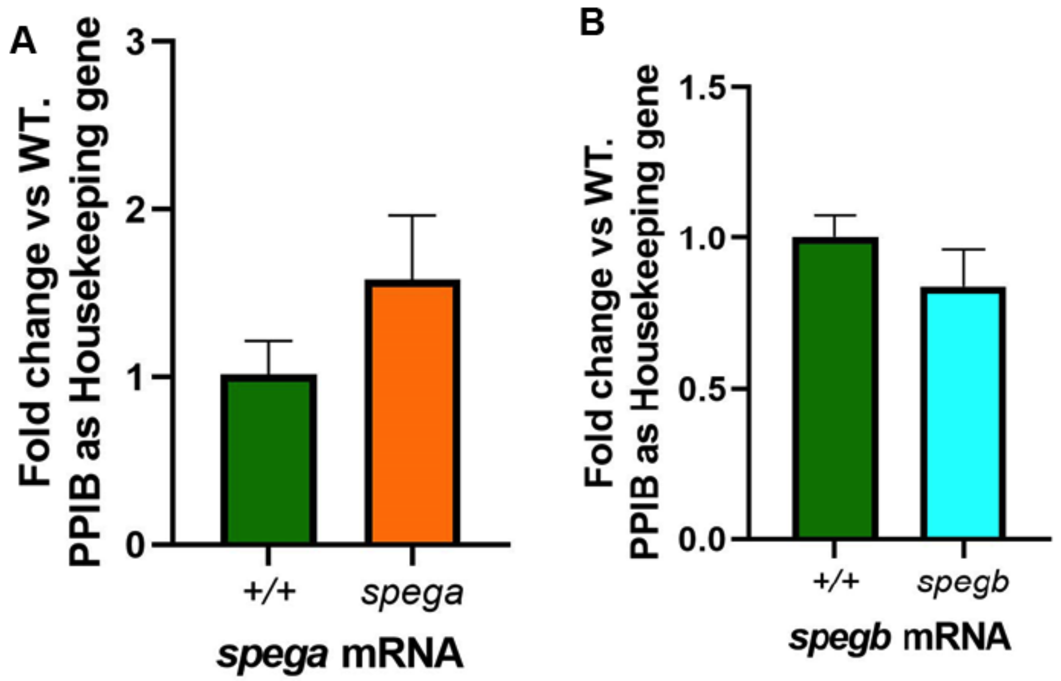

Fig. S2. RT-qPCR analysis on single mutants.

No significant changes were observed at 7 dpf for the levels of *spega* mRNA in *spegaΔ10*-KO (**A**), or *spegb* mRNA in *spegbΔ17*-KO (**B**). Levels of expression were first normalized to the housekeeping gene *ppib*, and then to WT. Results represent three independent experiments (technical triplicates per experiment). N=10-15 embryos per genotype per independent experiment. Columns and error bars represent Mean ± SEM. Two-tailed Student's *t*-test was performed: ns, not significant.

Disease Models & Mechanisms • Supplementary information

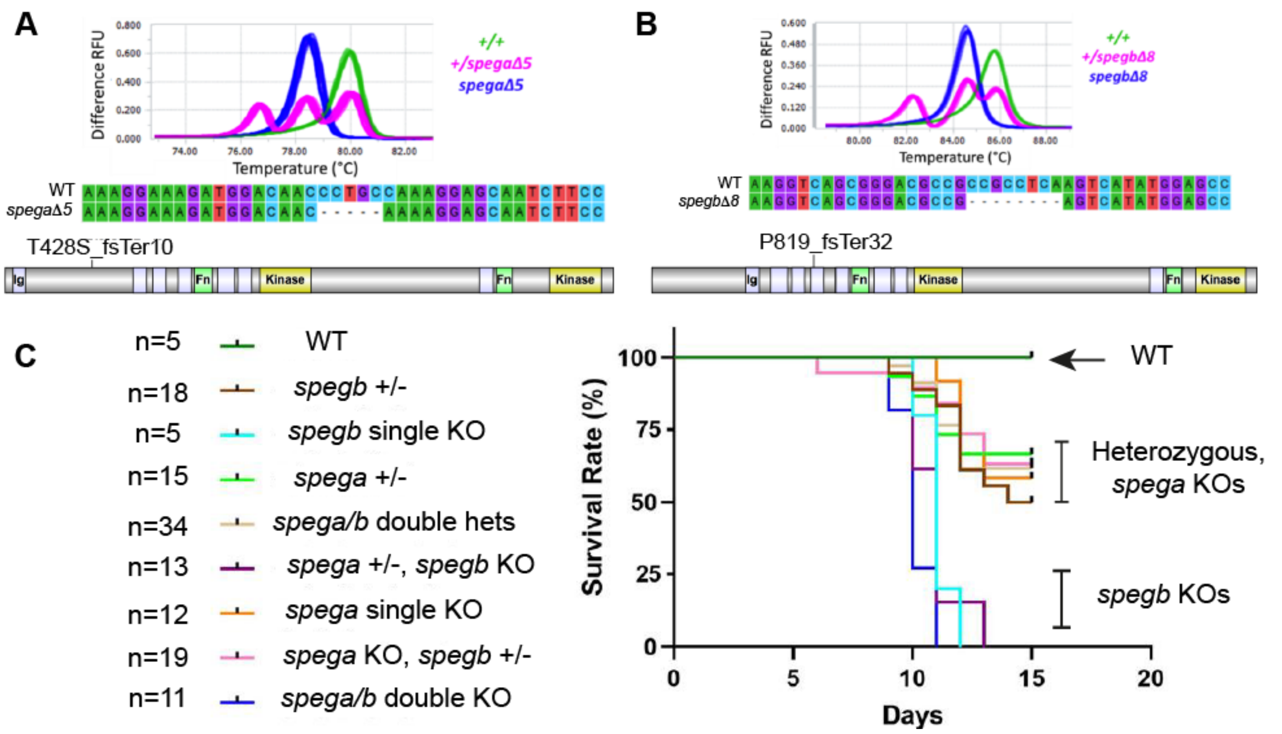

Fig. S3. Reduced survival in *spegaΔ5;spegbΔ8*.

Additional mutant lines were generated to validate the *speg*-DKO (*spegaΔ10;spegbΔ17)* survival phenotype. **(A** and **B)** HRM and Sanger sequencing confirmed the genotypes of (**A**) *spegaΔ5*, which carries a 5-bp deletion in exon 5 of *spega*, and (**B**) *spegbΔ8,* which carries an 8-bp deletion in exon 8 of *spegb.* Both are predicted to cause frame shift (fs) and premature stop codons. (**C**) Survival analysis across all *spegaΔ5* and/or *spegbΔ8* lines: survival was reduced in *spegb* KOs, *spega* +/-;*spegb* KO and *spega/b* double knockouts, with a median survival of 11, 11 and 10 dpf, respectively, and a maximum survival of 12, 13 and 11 dpf, respectively. This represents a significant decrease in survival as zebrafish can live up to 1.5-2 years (****P<0.0001), Mantel-Cox test. In contrast, WT and *spega* KOs have similar lifespans.

Disease Models & Mechanisms • Supplementary information

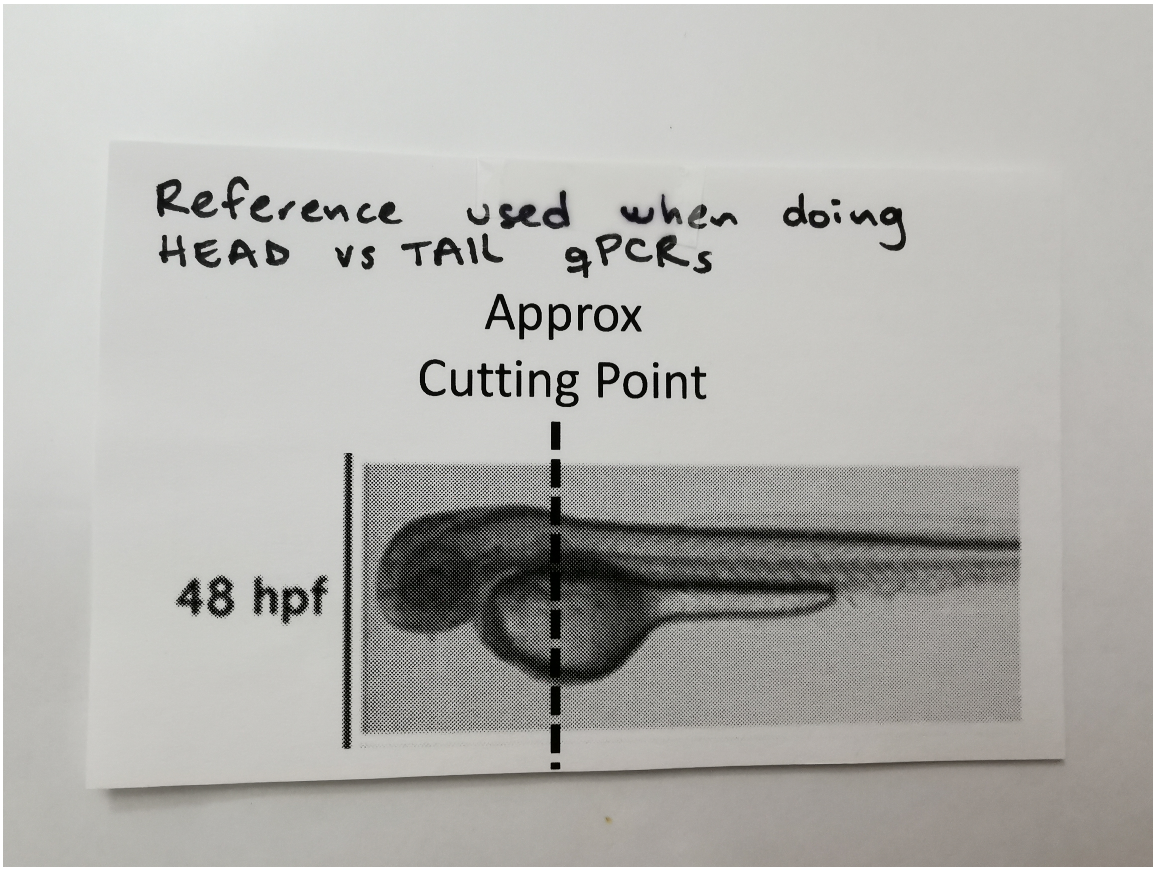

Fig. S4. An illustration showing the approximate cutting point (dotted line) to separate head vs tail for RT-qPCR analyses.
